# Anterior mediastinal large cell neuroendocrine carcinoma with elevated AFP: A case report and review

**DOI:** 10.3892/mco.2021.2248

**Published:** 2021-02-26

**Authors:** Justin Komisarof, Haoming Qiu, Moises J. Velez, Deborah Mulford

Mol Clin Oncol 14:Article no. 34, 2021; DOI: 10.3892/mco.2020.2196

Following the publication of this article, the authors have realized that a sentence in the Introduction was written incorrectly from the perspective of its comprehension. The sentence on the first page, right-hand column, lines 5-7, written as “When a diagnosis of LCNEC cannot be made…” should have been written as follows (changes highlighted in bold): “When a diagnosis of LCNEC cannot be made on a morphological basis, immunohistochemical markers exist that **may aid in the differentiation of** the disease from SCLC”.

The authors are grateful to the Editor of *Molecular and Clinical Oncology* for allowing them the opportunity to publish this corrigendum, and regret any confusion that the wording of this sentence may have caused for readers.

